# The French National Animal Health Surveillance Platform: an innovative, cross-sector collaboration to improve surveillance system efficiency in France and a tangible example of the One Health approach

**DOI:** 10.3389/fvets.2024.1249925

**Published:** 2024-08-21

**Authors:** Céline Dupuy, Célia Locquet, Christophe Brard, Laure Dommergues, Eva Faure, Kristel Gache, Renaud Lancelot, Alexandra Mailles, Justine Marchand, Ariane Payne, Anne Touratier, Aurèle Valognes, Sophie Carles

**Affiliations:** ^1^University of Lyon – French Agency for Food, Environmental and Occupational Health and Safety (ANSES), Laboratory of Lyon, Epidemiology and Support to Surveillance Unit, Lyon, France; ^2^French Ministry of Agriculture, Paris, France; ^3^Société Nationale des Groupements Techniques Vétérinaires (SNGTV), Paris, France; ^4^La Coopération Agricole, Paris, France; ^5^French Hunters' Federation (FNC), Issy-les-Moulineaux, France; ^6^GDS France, Paris, France; ^7^The French Agricultural Research and International Cooperation Organization (CIRAD), UMR Animal, Sante, Territoires, Risques, et Ecosystemes, Sainte-Clothilde, France; ^8^Animal, Sante, Territoires, Risques, et Ecosystemes, University of Montpellier, CIRAD, INRAE, Montpellier, France; ^9^Santé Publique France, Saint-Maurice, France; ^10^French Biodiversity Office, Paris, France; ^11^Association of French Managers of Public Veterinary Analysis Laboratories (ADILVA), Paris, France; ^12^French National Research Institute for Agriculture, Food and Environment (INRAE), Marcy-l'Étoile, France

**Keywords:** animal health, surveillance, epidemiology, One Health, collaboration, consensus

## Abstract

The French National Animal Health Surveillance Platform (NAHSP) was created in 2011. This network of animal health stakeholders was set up to improve surveillance efficiency for all health risks that threaten animal health, as well as zoonoses affecting human health. The NAHSP steering committee decides on the strategies and program of activities. It is composed of 11 institutions from both public and private sectors (policy-makers, scientific institutions, and representatives of farmers, veterinarians, hunters, and laboratories). A coordination team guarantees the implementation of the program and facilitates the activities of different working groups (WGs). Each WG is composed of technical experts with scientific, legal, and field knowledge from the sectors of animal health (livestock, companion animals, and wildlife), human health, and environmental health. Some WGs focus on a specific disease or health indicator, such as African swine fever or cattle mortality, while others cover cross-cutting topics, such as epidemic intelligence (EI), or specialize in aiding epidemiological investigations, such as the Q fever WG. The NAHSP stands out for its innovative approach because it is based on the concepts of consensus-building among participants, fostering collaboration, and embracing interdisciplinarity. Each proposal designed to improve surveillance is jointly developed by all the stakeholders involved, thereby ensuring its sustainability and acceptability among stakeholders. This process also has added value for decision-makers. As a pioneer platform, the NAHSP inspired the creation of two additional national surveillance platforms in 2018, one for plant health and the other for food chain safety. Both are organized in the same way as the NAHSP, which created a framework to place the emphasis on a One Health approach. For instance, four WGs are common to the three national surveillance platforms. This article aims to present this innovative approach to improve surveillance efficiency that could be of interest to other European countries or that could be rolled out at the European level.

## 1 Needs and challenges in animal health surveillance

The needs and challenges presented in this section are relevant for France but may also find relevance in other countries. Historically, most animal disease surveillance systems were designed as separate and stand-alone entities, with, for example, one system for a single species, sector, or notifiable disease. The interface between livestock and wildlife (e.g., organic farming, extensive farming, and urban farming), alongside the interaction between livestock/pets and humans (e.g., backyards), has increased over the past 20 years. This has led to increased disease transmission between these compartments, such as avian influenza and bovine tuberculosis (BT), highlighting the need to implement effective and integrated surveillance (1, 2). For instance, in France, certain diseases are monitored through various surveillance components involving different stakeholders. Influenza is monitored based on the assessment of the influenza virus in poultry, wild birds, swine, and humans. Bovine tuberculosis involves monitoring in cattle farms, at slaughterhouses, and in wildlife. Aujeszky's disease is monitored in swine farms, wild boars, and dogs. Integrating these components into a unified surveillance system for all diseases is challenging. This integrative thinking has been encouraged since the beginning of the 21st century through the One Health approach, but the main difficulty still lies in making the approach effective, sustainable, and efficient (3). Increasing the effectiveness of animal disease surveillance through an integrative approach helps identify and tailor more suitable prevention measures. It also helps prepare strategies for disease management or eradication.

Laboratory diagnostic methods are continuously improving, but the benefits are sometimes associated with more complex interpretations for epidemiologists. For instance, the interpretation of interferon-gamma (IFN-gamma) for bovine tuberculosis or polymerase chain reaction cycle threshold (PCR Ct) values for blue tongue requires laboratory expertise in addition to epidemiological knowledge. Improving a surveillance strategy involves having laboratory experts identify the most relevant methods to be used while considering their limitations to avoid misinterpretation. For instance, a positive bluetongue PCR result with high Ct values needs to be interpreted in light of the epidemiological context to discriminate between the active circulation of the virus and traces of earlier infection. The increasing complexity of the livestock sector, related to the international movement of animals, and the food and feed markets, means that more data are to be considered when working on animal disease surveillance. This also involves carrying out constant epidemic intelligence (EI) to help in assessing the risk of a disease being introduced into a country. Another aspect to consider is the diversity of stakeholders involved in surveillance, which include farmers, hunters, veterinarians, competent authorities, laboratories, among others. Their expectations and constraints are different and need to be taken into account when changes in the surveillance strategy are being considered, especially in the current context of financial limitations. Neglecting these considerations could lead to surveillance stakeholders finding the new strategy unacceptable and not implementing it as a result. These various aspects highlight the need to consider a variety of skills, such as field and laboratory expertise, risk assessment abilities, and knowledge of epidemiology, when working to improve surveillance effectiveness. All surveillance stakeholders should be involved in discussing topics of interest, understanding different perspectives, and reaching an agreement that maintains both scientific rigor and field pragmatism.

The rise in digitalization is helpful for gathering surveillance information and centralizing data. While financial support has been provided to develop new databases, it is far more difficult to obtain long-term human resources to analyze these data. For instance, in France, cattle mortality data have been used for syndromic surveillance (SyS) since 2013, but the sustainability of this approach is still challenging due to a lack of available and long-term human resources. The lack, or inappropriate timing, of data exploitation and feedback to surveillance stakeholders is one of the main issues in maintaining a high level of surveillance acceptability and stakeholder motivation. As an example, this has been demonstrated for bovine tuberculosis surveillance in wildlife in France (4). Finding new approaches to automatically and robustly produce relevant surveillance indicators with secure access is crucial; however, the indicators still need human interpretation.

Since the early 2010s, emerging animal diseases have been identified as a new challenge for disease surveillance (5). The outbreak of the Schmallenberg virus (SBV) in 2011, the COVID-19 pandemic in 2020, and the introduction of epizootic hemorrhagic disease into Europe in 2022 have highlighted the necessity to improve disease preparedness and response. Traditional surveillance approaches may not be sufficient in these cases. It is important to consider new methodologies, such as syndromic surveillance, and the research sector plays an essential role in identifying new methodologies to meet these needs. Implementing novel tools in practice remains challenging nonetheless. In addition, adopting a One Health approach is vital given the complexity of disease transmission, the ever-increasing movement of people, animals, and goods, and the increasing role of wildlife.

In light of these needs and challenges, a new approach was considered necessary. In 2010, stakeholders in France initiated a national brainstorming round table to suggest methods for achieving a paradigm shift. The subsequent action plan included the creation of a country-wide platform that became the National Animal Health Surveillance Platform, or NAHSP. This innovative approach is presented along with how it can provide a solution to meet these needs and challenges.

## 2 Development of the NAHSP

In 2011, the NAHSP was created to improve the efficiency of surveillance for all health risks that threaten animal health, as well as zoonoses that affect human health in France. The platform is a network of animal health stakeholders who work together to improve collaboration and increase efficiency. It is based on an agreement signed by the members of the NAHSP steering committee. Importantly, it is neither a legal entity nor a data-sharing platform.

The emergence of the Schmallenberg virus (SBV) in northern Europe in late 2011 provided the NAHSP with an opportunity to demonstrate its utility. A working group (WG) was rapidly established to propose a surveillance protocol to detect the potential introduction of SBV into France. This protocol was implemented by the French Ministry of Agriculture (MoA) at the beginning of 2012 and enabled the first cases to be detected at the end of January of the same year (6). A surveillance protocol was maintained until 2018, with the drafting of a surveillance report to support the competent authority's decisions in terms of surveillance and management of the disease (7). After this first successful proof-of-concept work, and after a few years of relevant activities with positive feedback from all stakeholders, the French MoA decided to extend the platform concept to plant health and food chain surveillance. The National Plant Health Surveillance Platform and the National Food Chain Surveillance Platform were thus created in 2018.

**Figure 1:**
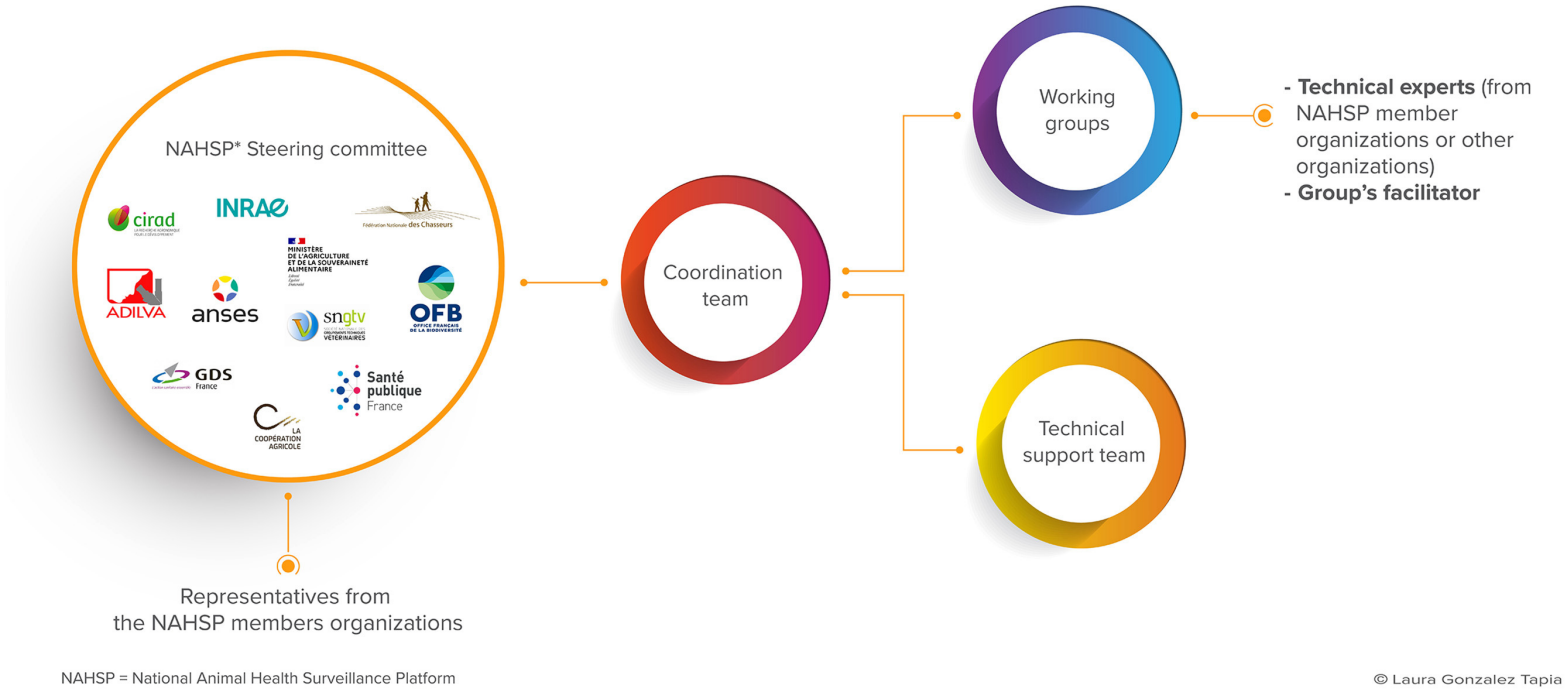
The NAHSP's organizational framework with its steering committee representing both the public and private sectors and its coordination team making the link between the steering committee, working groups, and the technical support team.

The governance of the NAHSP is overseen by a steering committee that is responsible for deciding on its strategies and program of activities. In 2023, the committee members represented 11 institutions from both public and private sectors: policy-makers, scientific institutions, representatives of farmers, veterinarians, hunters, and laboratories. These institutions are, in alphabetic order, mentioned in the following: ADILVA, an association of directors and managers of public veterinary analysis laboratories; ANSES, the French Agency for Food, Environmental, and Occupational Health & Safety; CIRAD, the French Agricultural Research Center for International Development; the French MoA; INRAE, the National Research Institute for Agriculture, Food, and the Environment; LCA, a federation of agricultural cooperatives; FNC, the national hunters' federation; GDS France, an animal health protection group; OFB, the French Biodiversity Office; SNGTV, a collective of veterinarians; and Santé Publique France, the National Public Health Agency (Figure 1). The NAHSP aims to support surveillance systems to meet their needs but does not make decisions regarding these systems. This decision process is attributed to surveillance system managers.

Several types of actions can be performed by dedicated working groups (WGs) to implement the NAHSP action plan, as mentioned in the following: (i) assist in the development and improvement of surveillance systems that the NAHSP supports; (ii) support the collection, analysis, and interpretation of surveillance data; (iii) facilitate communication of surveillance results, mainly through the NAHSP website or online restricted-access tools, to allow feedback to surveillance stakeholders and ensure suitable mainstream communication; (iv) support epidemiological investigations when cases are detected; and (v) perform national and international epidemic intelligence activities with both official and non-official data sources. Epidemic intelligence involves the collection, analysis, and interpretation of different sources of data to produce reports that are able to support decision-makers for further investigation and the prevention of potential health risks.

**Figure 2:**
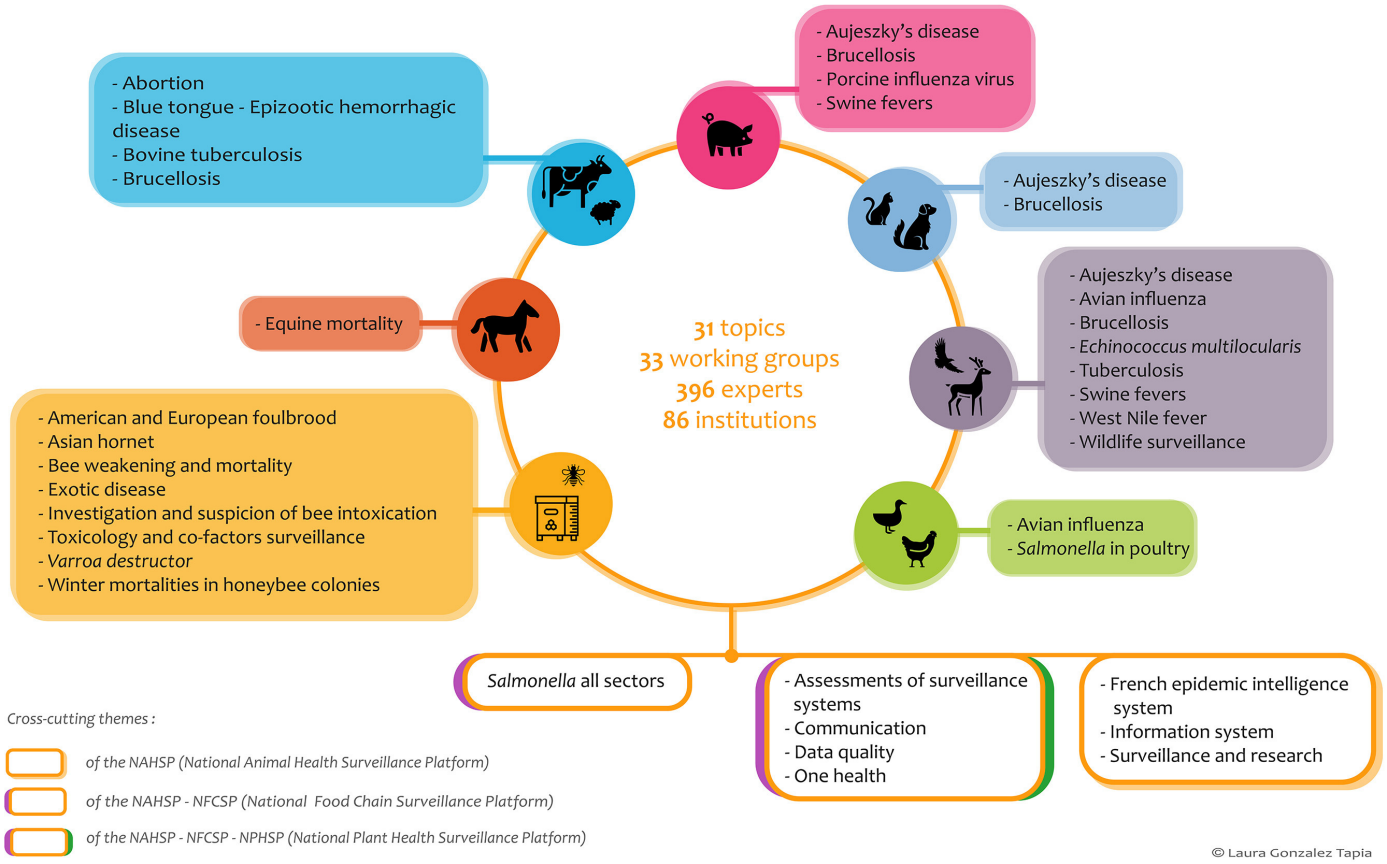
Topics of NAHSP working groups, which are composed of experts from animal health, human health, and environmental health sectors, depending on the scope of each WG.

Currently, a total of 33 WGs have carried out activities concerning 31 topics of interest in several different sectors of animal health: bees, cattle, horses, pets, poultry, small ruminants, swine, and wildlife (Figures 1, 2). Some WGs focus on a specific disease or health indicator, while others are cross-cutting or are dedicated to supporting epidemiological investigations (Figure 2). Each WG includes relevant technical experts with field expertise as well as scientific and legal knowledge from various animal health (livestock, wildlife, and pets), human health, and environmental health sectors (Figure 1). In 2023, 396 experts from 86 institutions were part of at least one of the NAHSP WGs. Their work is supported by a technical support team of 10 full-time equivalent staff members, eight of whom are long-term staff members with skills in epidemiology, statistics, information technology, or communication. An NAHSP coordination team of two full-time equivalent staff members ensures the implementation of the program, coordinates different WG activities, and manages the technical support team (Figure 1).

The platform is funded mainly by the French MoA, which covers the salaries of the technical support and coordination teams. An audit was carried out in 2021 and found an estimate of €3 million in annual funding for the three epidemiological surveillance platforms combined (8). Experts participate in WGs on a voluntary basis, constituting indirect financial support for the NAHSP from their employers. It was estimated in 2022 that this support amounted to six full-time equivalent staff members for 345 experts from 75 organizations. Importantly, actions are prioritized to match the human and financial resources available. The impact of any conflict of interest is limited by the diversity of experts within each group and the consensus approach, which enables unanimous agreement to be reached.

Three core values underlie the activity of all components of the NAHSP: consensus, collaboration, and a 2-fold multi-sectoral and multi-partnership approach. Suggestions for improving surveillance efficiency are put forward by WGs through a co-construction process and validated by consensus. This involves allowing sufficient time to listen to each stakeholder, to understand the expectations and constraints of the stakeholders, and to take scientific innovations into account. This process decreases the risk of rejection by any of the surveillance stakeholders and increases the robustness of a WG's suggested solution because all stakeholders are part of the proposal process.

The platform's organizational structure and the type of actions carried out were designed to address some of the challenges presented in the introduction of this article. The tangible examples developed in the following section demonstrate the platform's added value.

## 3 Added value and One Health approach

Detecting emerging diseases is challenging in many ways. The scientific community (9) posits that syndromic surveillance (SyS) appears to be increasingly relevant to such a program. Based on French cattle mortality data, Perrin et al. demonstrated the relevance of SyS both for the early detection of unexpected health events causing cattle mortality and for assessing health events with an impact on cattle mortality (10). In 2013, the French MoA asked the NAHSP to implement an operational SyS system based on the cattle mortality data. A dedicated WG was created to co-construct the future SyS system with data providers, competent authorities, field experts, including farmers and veterinarians, researchers, and data managers. A pilot phase of this SyS system, known as the syndromic bovine mortality surveillance sytem (OMAR) alert tool, was launched in 2018 in various local administrative regions to calibrate the thresholds to use based on the best trade-off between sensitivity and specificity for surveillance needs (11). These thresholds were revised twice before 2022, when they were accepted as final values in these local regions. This tool is used on an ongoing basis and has demonstrated its ability to detect signals not otherwise found by field actors. It enables early detection of events on farms and results in the early implementation of solutions to animal health and welfare issues that might otherwise lead to increased mortality. Moreover, the WG used the same data to develop additional tools that complement the SyS system. These tools are reports or dashboards that are automatically updated with indicators at the national, regional, and farm levels. They were designed to meet other needs identified by the WG experts for both animal health surveillance and animal welfare. For instance, dashboards were implemented to support the following: (i) competent authorities when performing risk-based inspections; (ii) farmers and veterinary associations to enable them to set up prevention actions for fellow farmers and veterinary associations; and (iii) rendering plants to facilitate monitoring activities. These complementary tools integrated the OMAR tool and are clearly considered positive collateral effects of the implementation of the SyS system. Interestingly, the same mortality data can be used for purposes other than the detection of emerging diseases, such as the detection of animal welfare issues. It is particularly challenging to maintain stakeholder motivation to interpret weekly alert reports that will probably not detect any emerging disease for a long period of time because such emergences are, by definition, unusual events. Finding complementary objectives for the SyS tool was, therefore, beneficial. A second positive effect of the OMAR project was to foster constant improvement in the quality of the mortality data in terms of completeness, robustness, and timeliness. This improvement is useful for any other project that exploits these data, regardless of the project's purpose. The next step will be to interpret alerts from the OMAR tool and the human health mortality SyS system to anticipate zoonotic diseases or factors influencing both animal and human health, such as climate phenomena.

The surveillance of bovine tuberculosis (BT) is based on several complementary surveillance systems: (i) active surveillance on the farm; (ii) systematic surveillance at the slaughterhouse; (iii) active surveillance when animals are moved; (iv) active surveillance in wildlife; and (v) passive surveillance in wildlife. Links between animal health and environmental sectors (wildlife) are thus essential to provide effective surveillance, taking into account this type of multi-host pathogen. These systems involve several stakeholders, some of whom are common to several of the systems, while others are only part of one system. Data from these surveillance systems were not initially centralized in the same database and were analyzed separately when used. Stakeholders from the livestock, wildlife, or slaughterhouse sectors did not usually process, analyze, or discuss their data together. Since its creation in 2011, the NAHSP has addressed the topic of BT to increase the efficiency of surveillance.

Two separate WGs were created in 2011, one dedicated to on-farm BT surveillance, named the BT WG, and the other dedicated to BT surveillance in wildlife, named the Sylvatub WG. These two WGs were linked together from the start through the participation of the coordinator of one group as an expert in the other group and vice versa. A single group would have been very large, potentially leading to difficulties in working efficiently. Over several years, each group took the time needed to learn how to work collectively, considering the diversity of expertise within each group, and conducted its work plan in its own area, i.e., to improve its surveillance effectiveness via indirect information gathering through each WG coordination team. Strong working relationships were developed over time, with co-constructed surveillance zoning taking into account both the farm and wildlife surveillance indicators. The NAHSP technical support team helped these WGs to centralize, clean, and analyze their data. A restricted-access dashboard dedicated to Sylvatub was implemented to share the surveillance indicators and their representation in a secure yet user-friendly way with local and national surveillance coordination teams. Since 2019, an annual publication common to both BT and Sylvatub WGs has been published in a national epidemiological journal (12, 13). The publication is co-constructed with experts from both groups. Since 2022, common data analysis reports have been produced by the NAHSP technical support team as input for these WGs, stimulating ideas on how to improve surveillance effectiveness and as support material for the competent authority (MoA), which is required to forward official indicators to the European Food Safety Authority. The automation of the data analysis process has saved time and increased data quality. This automation process is carried out as soon as it has been identified as relevant and efficient for any of the NAHSP topics.

Although data related to outbreaks detected through slaughterhouse surveillance were taken into account by the BT WG, there was no expert from the slaughterhouse sector in this WG. Consequently, actions to improve BT surveillance in slaughterhouses were not taken into consideration by this WG. In 2020, a third WG dedicated to BT was created to focus on BT slaughterhouse surveillance. The coordinator of the BT WG is part of the coordination team of this BT slaughterhouse WG. Slaughterhouse data analysis was automated, and a user-friendly dashboard was created, with the support of the NAHSP technical support team. A step-by-step approach was chosen to gradually increase the integrative approach to bovine BT surveillance. These WGs are also a place where experts can regularly discuss ideas and feel free to exchange their points of view on the situation of BT surveillance, even when there are no outbreaks. These discussions help facilitate communication in times of crisis, as the same stakeholders are involved and have already been working together for a long time. This is a highly valuable positive collateral effect of all NAHSP WGs.

Animal health surveillance cannot be considered a stand-alone unit due to globalization, which leads to an international movement of animals, food, feed, and people. Importantly, globalization is associated with an ever-increasing risk of new diseases or health threats being introduced into a country, an aspect that must be considered by both competent authorities and all surveillance stakeholders. In this regard, the NAHSP was tasked with developing a national and international epidemic intelligence (EI) project. Since the creation of the NAHSP in 2011, a dedicated epidemic intelligence WG has been set up. This WG includes representatives of epidemic intelligence end users (competent authorities and representatives of farmers, veterinarians, hunters, and laboratories), researchers of EI methodology, and epidemiologists (14). Official and non-official data are analyzed by the NAHSP technical support team (1.5 full-time equivalent staff members). An EI editorial board, comprised of the competent authority and epidemiologists, meets weekly as it is responsible for producing EI publications. This editorial board relies on its national and international network of experts to complement its interpretation. Weekly, seasonal, and “breaking news” reports are generated and made public through the NAHSP website (15). The EI WG has confirmed the value of EI publications in increasing the awareness of surveillance stakeholders and helping prevent diseases from entering France. The NAHSP EI process has gradually evolved in several ways. An increasing number of data sources are considered for EI production through the support of researchers, with, for instance, the implementation of a tool known as “Padiweb” for media data analyses (16). This tool has been in routine use since 2022. More epidemiologists have joined the editorial team for its weekly meetings (from three people in 2011 to 14 people in 2023). This increases the robustness of interpretation through multiple viewpoints and helps identify additional experts to contact as needed to investigate certain signals in more depth.

For zoonotic diseases, such as West Nile (WN) fever, both animal health data and human health data have been considered for the creation of the EI team. Before 2020, only animal health experts were involved in the interpretation. Since 2020, human health experts have also been involved in the development of the WN seasonal report, and since 2022, for WN weekly reports. During the SARS-CoV-2 period, the EI teams monitored animal cases through a dedicated report updated 13 times from April 2020 to February 2022. Similarly, for WN, the SARS-CoV-2 report was also produced initially with a team of animal health experts and then extended to include human health experts. Data analyses within the EI team were also improved over time, from manual analysis based on Excel files to automated data analyses using R. This has made it possible to save time while increasing quality. These examples demonstrate the NAHSP's ongoing improvement process and step-by-step approach when implementing improvements.

Concerning the measurement of success, there are no formalized indicators on the usefulness or success of the work of the NAHSP but there are several examples of successful projects. Each year, several national regulations are issued or amended by the MoA based on work produced by the platform's WGs. Examples include surveillance of bovine tuberculosis, avian influenza, and blue tongue. Dashboards are used by surveillance stakeholders both during peacetime between outbreaks and during emergencies, receiving positive feedback (tuberculosis, avian flu, epidemic intelligence, etc.). The data analyses performed are used by the national competent authority to submit official indicators to the European Food Safety Authority or European Commission (tuberculosis, blue tongue, salmonella, etc.). The time required to analyze surveillance data has been greatly optimized by both the automation of data analysis and the improvement of the data collection process. For instance, while 1,600 h were needed to analyze bovine tuberculosis data in 2019, only 300 h/year have been required since 2022.

## 4 Constraints and challenges

Since 2011, stakeholders involved in the NAHSP, from steering committee members to WG experts, have been satisfied with what was considered an innovative approach with shared governance between the public and private sector and a co-construction method based on the principle of consensus. However, managing this organization presents challenges in several aspects.

First, sustainable financial resources are difficult to find. Since its creation in 2011, the main source of funding has been the public sector, i.e., the French MoA, which has funded the human resources needed for coordination and technical support teams. Since 2011, the NAHSP has demonstrated its usefulness and has been receiving an increasing number of requests to address issues within the scope of surveillance, even as public funding has been decreased. There is, thus, a discrepancy between its objectives and the means available to achieve them, considering the challenge of ensuring sustainable financial support. Exploring other sources of funding that comply with keeping a not-for-profit approach while remaining independent from potential private financial support will be necessary. This constraint also highlights the need to prioritize actions, which is one of the tasks of the steering committee.

Second, the benefits of developing long-term WGs have already been described, but it has proven difficult to maintain WG organization and facilitation over time. Without a dynamic and committed team of experts in the appropriate field to lead the WG, it cannot operate correctly. In this regard, staff turnover can affect WG coordination. To limit this risk, it is preferable to set up an internal WG coordination team when possible. Another challenge with long-term WGs is to maintain experts' motivation to contribute as these contributions are made on an individual and voluntary basis and depend on the parent institution's willingness to grant experts sufficient time. Therefore, it is essential that experts benefit from their participation in WGs, for example, through network-building and information exchange. They also need to be aware that their contribution to the improvement of surveillance efficiency in practical ways is of great value.

Finally, although the NAHSP has improved its One Health approach over time, much work remains to be done. New environmental and climate concerns need to be better integrated and addressed. This is particularly important for topics related to bee health and vectorial disease surveillance, but these concerns should be considered for many topics in animal health. Looking to the future, new methodological approaches should be investigated to address this challenge effectively, which will, in practice, lead to a broadening of the scope of expertise represented in existing WGs.

## 5 Lessons learned

Over 10 years of operation, the NAHSP has demonstrated its value in supporting managers in the surveillance of animal health and disease to improve surveillance efficiency. Several examples of this are described in Section 3. The NAHSP has adopted the One Health philosophy over time through different approaches. Based on the platform's decade of experience, it appears that an incremental approach with a commitment to continuous improvement is a good strategy for building a solid and consistent One Health approach. The membership of the steering committee has also evolved over the years. The environmental sector was included via the French Hunters' Federation and the French Biodiversity Office in 2013, and then the human health sector was included via the National Public Health Agency in 2022. Experts from these institutions were already participating in some of the WGs before they became members of the steering committee, but this was a step toward closer cooperation. Similarly, the same approach was applied to the WGs. Since 2011, a swine flu WG has focused on supporting the RESAVIP network, a surveillance system that monitors the swine influenza A virus. Its goal is to describe the virological and epidemiological patterns of the virus and to detect the occurrence of new patterns that could have an impact on animal or human health (17). Until 2021, this WG included only animal health experts. However, it was decided that a human health expert would be invited occasionally, when information related to the impact of the virus on human health was discussed. After a year, initial feedback revealed that this was not an appropriate way of operating, primarily due to a large number of gaps in meeting invitations. In 2022, it was therefore decided to systematically invite the human health expert. The following year, it was further decided to include this expert in the WG. The same step-by-step approach was implemented for the WG investigating Q fever. Naturally, a certain amount of time was needed for animal health stakeholders to learn how to work together (2019–2021), after which the group added two experts from the human health sector, who have been part of the WG since 2022.

To build a One Health approach in the same incremental manner, interactions between WGs on the same topic can gradually increase to allow each WG to take shape and then develop new cooperative relations between existing WGs. The BT WGs illustrate this approach effectively (see Section 3).

In 2018, a new step in improving the One Health approach was achieved when two more surveillance platforms were set up, one on plant health and the other on the food chain. A coordination group common to both platforms was created simultaneously and was composed of each platform's coordination team. This group provided a suitable framework for exchanges and for identifying joint work. Since 2018, five WGs common to two or three platforms have been created (Figure 2). One WG is dedicated to a zoonotic disease (*Salmonella*), while the others are dedicated to methodological topics.

These examples show that there was no magic recipe for the NAHSP's implementation of a successful One Health approach. Each situation needed a tailored strategy. For WGs, the best way was found to be through co-construction with WG experts, keeping in mind the need for flexibility, because one set-up may meet the requirements at one point but a different set-up may be best when other experts are involved. The key is to enable constant reevaluation. It has, however, become clear that it is easier to develop a One Health approach between crises or outbreaks than during crises because experts need to be given time to develop working relationships and mutual understanding. The NAHSP aims to implement a sustainable One Health approach, and as such, WGs are designed to be long-term groups. Unsurprisingly, short-term WGs have rarely been created.

The “platform concept” based on consensus, collaboration, and a multisectoral and multi-partnership approach can be applied to other fields. The extension of the concept in France from animal health to plant health and food chain safety demonstrates this principle. Although our examples relate to France, this concept could be applicable in other countries, provided there are similar needs identified.

## Data Availability

The original contributions presented in the study are included in the article/supplementary material, further inquiries can be directed to the corresponding author.
